# Correlation Between Body Mass Index and Apnea-Hypopnea Index or Nadir Oxygen Saturation Levels in Patients With Obstructive Sleep Apnea

**DOI:** 10.7759/cureus.59066

**Published:** 2024-04-26

**Authors:** Ahmed Uzair, Muhammad Waseem, Aun Bin Shahid, Nauman I Bhatti, Muhammad Arshad, Asher Ishaq, Muhammad Sajawal, Zoha Toor, Osama Ahmad

**Affiliations:** 1 Pulmonary Medicine, Sahiwal Medical College & Allied Teaching Hospital, Sahiwal, PAK; 2 Internal Medicine, Sahiwal Medical College & Allied Teaching Hospital, Sahiwal, PAK; 3 Internal Medicine, King Edward Medical University & Allied Hospital, Lahore, PAK; 4 Emergency Department, Pak Red Crescent Medical College & Allied Teaching Hospital, Lahore, PAK; 5 Medical Intensive Care Unit, Mukhtar A. Shiekh Hospital, Multan, PAK; 6 Internal Medicine, Abwa Medical College Faisalabad, Pakistan, Faisalabad, PAK

**Keywords:** sleep apnea syndromes, oxygen saturation, hypopnea, body mass index, apnea

## Abstract

Background: Apnea-hypopnea index (AHI) and nadir oxygen saturation (SpO2) are the indexes used to measure the severity of obstructive sleep apnea (OSA). Obesity, measured by body mass index (BMI), is one of the main contributing factors to the onset and severity of OSA in patients. This study was conducted to find the association between BMI and OSA severity indexes, mainly AHI and nadir SpO2 levels.

Methods: Polysomnography reports of patients with diagnosed OSA in a teaching hospital were retrospectively reviewed. BMI, AHI, and nadir SpO2 levels were recorded from the sleep study reports of the patients. Spearman’s Rho test was applied to find the correlation between BMI and AHI/nadir Spo2 levels.

Results: A total of 167 patients were included in the study, comprising 83 males and 84 females. The Mann-Whitney U test was utilized to investigate the association between BMI and gender and age groups. The analysis revealed a significant difference in BMI between males and females, with females having a higher BMI. However, there was no significant difference in BMI among individuals in the early middle and late middle age groups. Spearman’s Rho test was employed to explore the correlation between BMI and AHI/nadir SpO2 levels. The results indicated no significant correlation between BMI and AHI (*p *= .122) or nadir SpO2 levels (*p *= .239).

Conclusion: Contrary to common belief, BMI was not linked to the severity of OSA. It implies that several other factors, independent of BMI, play a role in the disease progression and severity.

## Introduction

Obstructive sleep apnea (OSA) has emerged as a common sleep disorder caused by intermittent and repetitive episodes of complete or partial upper airway obstruction during sleep, resulting in hypoxia and fragmented sleep [1]. This results in significant daytime symptoms, including excessive sleepiness, fatigue, tiredness, mood change, poor memory, and a lack of concentration. Various anatomical and neuromuscular variables interplay in the etiology of OSA, resulting in upper airway collapse during sleep [2]. The pharyngeal dilators maintain the patency of the upper airways, which relax during sleep. Some narrowing of the pharyngeal dilators is normal at sleep, but excessive narrowing can occur due to small pharyngeal size when awake; the reasons include infiltration of fat within the pharyngeal tissues, external compression from neck fat, or enlarged tonsils [3]. Narrowing of the airways at sleep onset is also a contributing factor that may be caused by pressure symptoms from a mass, neuromuscular disease with pharyngeal involvement, muscle relaxants, sedatives, alcohol, and increasing age.

OSA is caused by a variety of risk factors, ranging from obesity to lifestyle variables like alcohol intake and smoking [4]. Two key parameters are essential in determining the degree of severity of OSA: the apnea-hypopnea index (AHI) and the nadir oxygen saturation (Nadir SpO2), both of which can be measured by overnight polysomnography, more commonly known as a sleep study [5,6]. AHI implies the number of apneas and hypopneas in one hour of sleep. OSA can be graded by the value of AHI as follows: AHI ˂5 is normal, AHI 5-15 is mild OSA, AHI 15-30 is moderate OSA, and AHI ˃30 is severe OSA. Nadir SpO2 represents the lowest oxygen saturation level reached during sleep, indicating the degree of nocturnal hypoxemia. These parameters not only provide the clinical diagnosis and severity indexes of OSA but also mark the importance of addressing oxygen deficiency as an essential aspect of patient outcomes [7].

Body mass index (BMI) has been shown to significantly impact OSA, with obesity as one of the leading risk factors [8]. Increased BMI increases the risk of developing OSA, exacerbating the negative effects of the condition. Understanding the complex relationship between the severity of OSA and BMI is important for planning interventions that consider the unique needs of each patient, regardless of their BMI category [9,10].

This retrospective study investigates the correlation between BMI and two important OSA severity indexes, AHI and nadir SpO2 levels. By defining the correlation between these entities, we aim to shed light on the trends that help our understanding of OSA pathophysiology and facilitate the development of targeted treatment guidelines.

## Materials and methods

Sample size

The sample size was determined using OpenEpi software (Version 3.01, Open Source Epidemiologic Statistics for Public Health, www.OpenEpi.com), with a population size set at 1 million and a reported OSA frequency in the literature of 12.4% [11]. A confidence limit of 5% was applied, resulting in a calculated sample size of 167. Patient records from the Pulmonology Department of Sahiwal Teaching Hospital, Sahiwal, Punjab, Pakistan, specifically those advised for polysomnography from November 2018 to November 2023, were acquired and examined for inclusion criteria. Inclusion criteria encompassed individuals aged up to 60 years and confirmed diagnoses of OSA. Exclusion criteria involved patients with a history of smoking, alcohol intake, and other pulmonary comorbidities.

Definitions

BMI was defined as a patient’s weight in kilograms divided by the square of height in meters [12]. Apnea was the reduction in the airflow by >90% for >10s during sleep. Hypopnea was defined as a reduction in the airflow >30% associated with either oxygen desaturation or arousal during sleep [13]. AHI was the number of apneas and hypopneas divided by the total sleep time. Nadir SpO2 was the lowest value of SpO2 during the sleep study. OSA was graded as follows: AHI >5 is normal, AHI 5-15 is mild OSA, AHI15-30 is moderate OSA, and AHI > 30 is severe OSA [14].

Statistical analysis

The statistical software Statistical Package for Social Sciences (SPSS), version 27.0 (IBM Corp., Armonk, NY) was utilized for the statistical analysis. Descriptives of the baseline variables were obtained, and the Kolmogorov-Smirnov test of normality was applied as the sample size was greater than 100 (n=167). The Mann-Whitney U test was performed to find the association of BMI with gender and the age groups divided as early middle age (35-44) and late middle age (45-60). The correlation between the BMI and AHI/nadir SpO2 levels was tested using Spearman’s Rho test.

## Results

Descriptive statistics of the baseline variables were calculated and reported (Table 1). The Kolmogorov-Smirnov normality test was applied, revealing that only BMI was normally distributed (p=0.06).

**Table 1:** Descriptive analysis. *p-*value considered significant at <.05; mean with SD for parametric variable and median with IQR for non-parametric variables have been reported. SD = Standard deviation; IQR = Interquartile range; BMI = Body mass index; AHI = Apnea-hypopnea index, SpO2 = Oxygen saturation.

Variables	Mean	SD	Median	IQR	p-value
Age	-	-	47	11	< .001
BMI	38.7	7.4	-	-	.060
Min SpO2	-	-	66	21	< .001
Max SpO2	-	-	99	02	< .001
AHI	-	-	34.1	44.9	< .001

**Figure 1:**
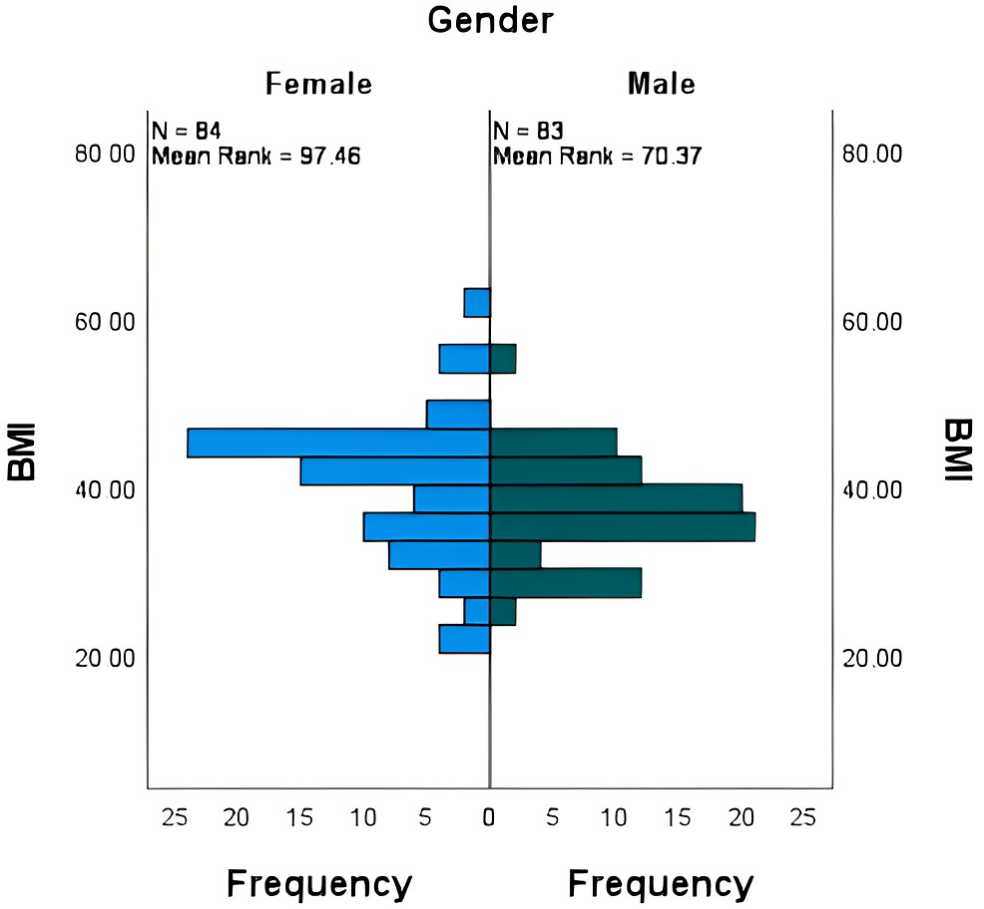
Association of BMI with gender. BMI = Body mass index.

**Figure 2:**
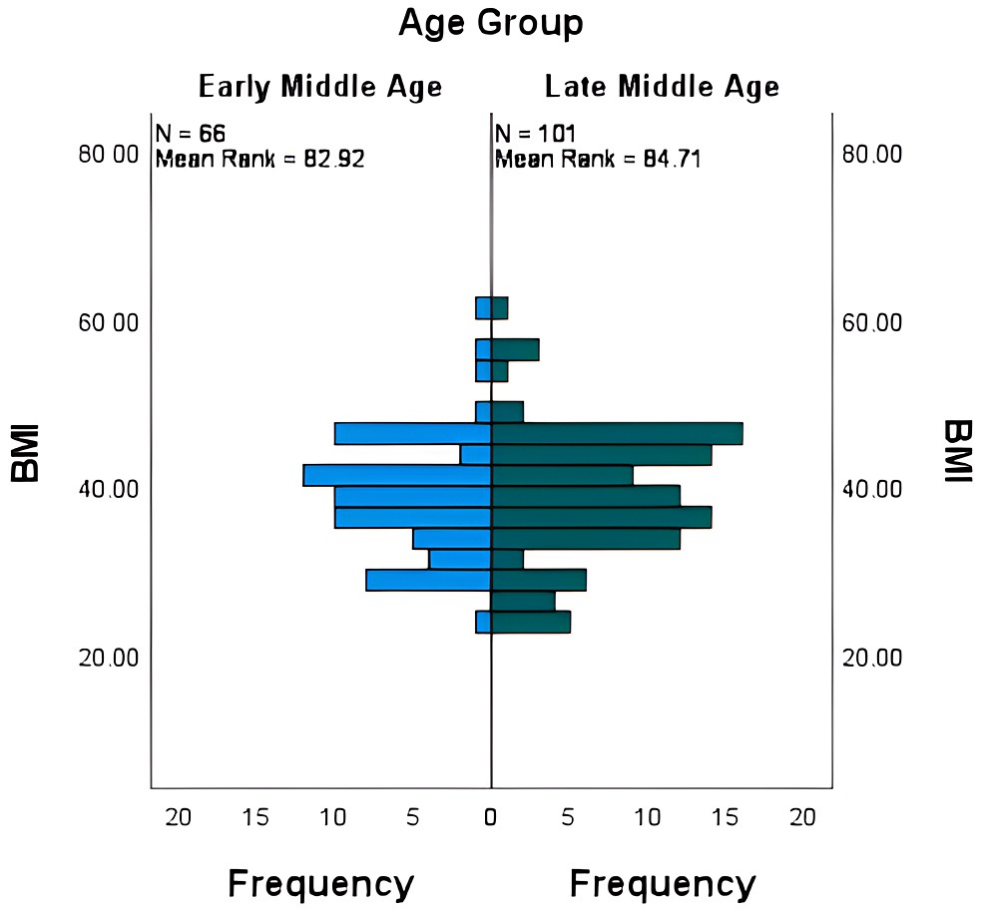
Association of BMI with age groups. BMI = Body mass index.

The Mann-Whitney U test was used to evaluate the differences in BMI by gender and age group. The test revealed that there is a significant difference in BMI between genders (with females having a higher BMI than males) leading to the rejection of the null hypothesis that states there is no difference in BMI between males and females, as shown in Figure 1. Conversely, no significant difference in BMI was observed between the two age groups (early middle age and late middle age), and thus the null hypothesis that there is no difference was accepted, as evidenced in Figure 2.

**Figure 3:**
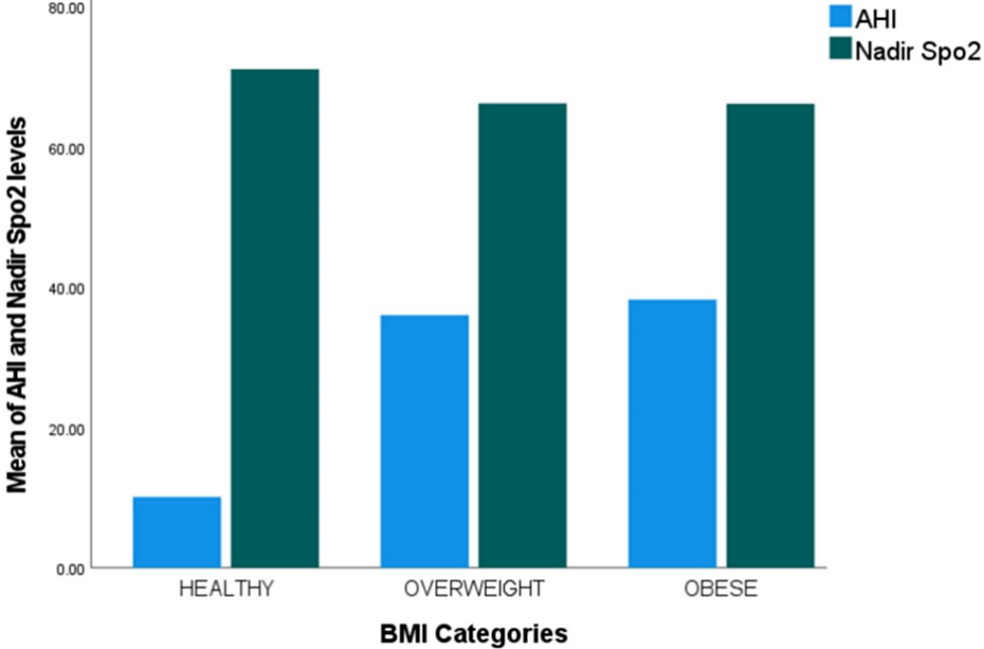
Bar chart showing various BMI categories with associated mean AHI and nadir SpO2 levels. BMI categories: Healthy if BMI is 18.5 to <25; overweight if BMI is 25 to <30; obese if BMI is >30. BMI = Body mass index; AHI = Apnea-hypopnea index; Nadir SpO2 = Nadir oxygen saturation.

To investigate the relationships between BMI and AHI/nadir SpO2 levels, the Spearman’s Rho test was applied. This test revealed no significant correlation between BMI and AHI or nadir SpO2 levels. Consequently, the null hypothesis that there is no correlation between these variables was accepted, and the results are outlined in Table 2 and Figure 3. However, a significant negative correlation was found between AHI and nadir SpO2 levels. In summary, the reported analysis suggests that while BMI differs significantly between genders, it doesn't vary between certain age groups, and no significant correlation exists between BMI and the severity of obstructive sleep apnea or the lowest oxygen saturation levels.

**Table 2:** Spearman’s Rho correlation analysis. Correlation coefficient (p-value) ** p ≤ .01. BMI = Body mass index; AHI = Apnea-hypopnea index; Nadir SpO2 = Nadir oxygen saturation.

	BMI	AHI	Nadir SpO2
BMI	1	.12 (.122)	- .08 (.289)
AHI	.12 (.122)	1	- .58**(˂ .001)
Nadir SpO2	- .08 (.289)	- .58**(˂ .001)	1

## Discussion

This retrospective study offers insight into the correlation between BMI and AHI/nadir SpO2 in patients diagnosed with obstructive sleep apnea (OSA). The complex interactions between BMI, gender, age, and OSA severity are highlighted by the analysis of 167 patients [15]. The study noted a significant difference in BMI between male and female patients. Utilizing the Mann-Whitney U test, it was found that BMI was significantly higher in women than in men. This observation is based on the literature written about gender differences in body composition and distribution of fat, which may play a role in variations of the severity index and susceptibility to OSA [16].

In contrast, no statistically significant differences were found between the BMIs of individuals in the early middle and late middle age groups when examining BMI across the two defined age groups. This proposes that age may not be the predominant factor influencing the variation in BMI among OSA patients within the range of this study. A more generalized analysis could be obtained from future studies considering a wide range of age groups.

Going on to the main objective of this research, Spearman's Rho test was utilized to find the correlation between BMI and AHI/nadir SpO2 levels. Remarkably, according to the results, neither the AHI nor the nadir SpO2 levels were significantly correlated with BMI. These study results are in contrast to some studies published earlier. Peppard et al. (2000) conducted a follow-up study on patients diagnosed with OSA, in which they reported a 32% increase in AHI with a 10% increase in body weight [17]. Friedman et al. (1999) reported a positive correlation of BMI with respiratory disturbance index, modified Mallampati score, and tonsil size [18]. In another study by Schäfer et al. (2002), a linear correlation of AHI with BMI, fasting blood sugar, fibrinogen level, and uric acid was observed [19].

However, a few studies are based on the results we have provided. Ciavarella et al. (2018) found no significant correlation between BMI and AHI, but a significant correlation between BMI and nadir SpO2 was observed [20]. Pendleybury et al. (1997) and Sforza et al. (1993) conducted a follow-up study to document the changes in baseline variables and their effect on OSA severity indexes, in which they found no significant correlation of BMI with AHI or nadir SpO2 levels [21,22].

This suggests that within the scope of this investigation, differences in BMI were not a reliable indicator of the severity index of OSA, as measured by AHI, or the corresponding oxygen desaturation, as indicated by nadir SpO2 levels. The commonly accepted belief that a higher BMI is directly associated with a higher severity of OSA is questioned by the need for a significant correlation. This study stands out as it raises doubts about the validity of BMI as the sole measure of OSA severity. This study, which denotes the association between BMI and OSA severity indices, shows that a strong correlation is required to support the more widely accepted theory about this relationship. This deviation from conventional literature highlights the nuanced relationship between OSA severity and BMI and calls for more research on the multidimensional nature of OSA pathophysiology such as the anatomy of the upper airways, genetic predispositions, and lifestyle choices.

The anatomy of the upper airways plays an important in the pathophysiology of OSA. While increased BMI or obesity is a well-established risk factor due to increased deposition of soft tissues in the upper airway, it is not the only determinant of the degree of air way collapse. Independent of BMI, changes in craniofacial morphology, such as a recessed chin, enlarged tonsils, or a thin or retrognathic jaw, can considerably contribute to airway blockage. This study marks many situations in which people with apparently normal BMIs have physical characteristics that make them more susceptible to OSA, thus supporting the idea that BMI is not the only risk factor responsible for the severity of OSA. Also, Stradling et al. (1991) have concluded that neck and abdominal fat, and neck circumference were effective in predicting the OSA severity rather than BMI, thus further strengthening the findings of this study [23].

Exclusive of the BMI, genetic changes, influencing the tone of the upper airway muscles, the ventilatory control mechanisms, and the shape of the craniofacial region can also modify the vulnerability to OSA. Moreover, changes in lifestyle, especially weight gain or reduction, might drastically affect the severity of OSA over time, making it more ambiguous to understand the association between BMI and AHI or nadir SpO2 levels. While weight management programs are routinely advised as the initial management of OSA, not all patients have appreciable decreases in their AHI even after losing weight. Additionally, lifestyle choices like smoking, drinking, or a sedentary lifestyle can also make OSA symptoms worse, independent of the BMI.

Although this study sheds light on the relationship between BMI and OSA, its retrospective design and narrow age range may limit the external validity of this study. Due to the retrospective approach, the study's capacity to outline the link between factors may have been compromised by its incapacity to prove causation. Moreover, the narrow range of age of this research limits the applicability of its conclusions to a wider window of demographics. The severity, prevalence, and association between BMI and OSA may vary in different age groups, making the conclusions less relevant to people beyond the designated age range. Moreover, age-specific factors that could affect the severity and risk of OSA, present a chance for additional improvement. Further advances in studies with a more extensive range of demographic variables and more thorough patient profiles covering wider age groups in the future may improve our knowledge of the complex relationships among BMI, gender, age, and OSA severity. These studies would be pivotal in improving risk assessment models and developing specialized treatment plans for OSA patients.

## Conclusions

Previous studies have suggested that obesity, as measured by body mass index, is a major risk factor for the development and severity of obstructive sleep apnea. However, recent research has challenged this notion and found no significant correlation between BMI and the severity indexes of OSA. This suggests that other factors influencing the progression and severity of OSA are unrelated to BMI. In addition to BMI alone, other contributing factors should be considered in future research when examining alternative determinants of OSA severity. These factors may include craniofacial anatomy, hormonal imbalances, genetic predisposition, upper airway muscle function, and other underlying medical conditions. Furthermore, it is essential to explore the impact of lifestyle habits such as smoking, alcohol consumption, and physical activity on the severity of obstructive sleep apnea. By considering a more comprehensive range of factors, researchers can better understand the complex mechanisms underlying OSA and develop more effective treatment and management strategies.
